# Synergistic Impact of Graphitic Carbon Nitride Supported Pd–Cu Bimetallic Nanoparticles for Direct Ethanol and Methanol Fuel Cell Applications

**DOI:** 10.1021/acsomega.5c06123

**Published:** 2025-11-17

**Authors:** Pariksha Bishnoi, Nirankar Singh, Samarjeet Singh Siwal, Vijay Kumar Thakur

**Affiliations:** a Department of Chemistry, M.M. Engineering College, 75850 Maharishi Markandeshwar (Deemed to be University), Mullana-Ambala, Haryana 133207, India; b Biorefining and Advanced Materials Research Center, 3123 Scotland’s Rural College (SRUC), Kings Buildings, West Mains Road, Edinburgh EH9 3JG, U.K.

## Abstract

Direct ethanol fuel cells (DEFCs) hold significant promise as sustainable energy conversion devices, yet the slow kinetics of ethanol (EtOH) oxidation remain a critical challenge. In this study, we present a novel catalyst comprising palladium–copper (Pd–Cu) bimetallic nanoparticles (NPs) based on graphitic carbon nitride (gC_3_N_4_) as an effective anode catalyst toward EtOH and methanol (MeOH) electrooxidation. The Pd–Cu/gC_3_N_4_ catalyst was synthesized via a facile and scalable technique, showing high catalytic performance and stability toward EtOH and MeOH electrooxidation. The enhanced efficiency is due to the gC_3_N_4_ support, which provides uniform dispersion and effective charge transfer; the Cu shows a bifunctional effect, which supplies oxygenated species to eliminate intermediates. The electronic interaction between Pd and Cu enhances the ethanol oxidation kinetics. Synergistic effects can explain the improved catalytic behavior. Electrochemical characterization, including cyclic voltammetry and chronoamperometry, demonstrated the superior performance of the Pd–Cu/gC_3_N_4_ catalyst compared to conventional catalysts (such as Pt/C or Pd/C etc.), ascribed to the synergetic consequence among Pd and Cu NPs and the superior catalytic activity and unique electronic property of gC_3_N_4_ support. To examine the proposed material’s unique properties and superior catalytic performance, its performance was compared with gC_3_N_4_, used as a reference material. The Pd–Cu/gC_3_N_4_ shows better current density (CD) values with higher forward current peak maxima for 1 M EtOH (4.54 mA/cm^2^) and 1 M MeOH (22.16 mA/cm^2^) in the presence of 0.5 M KOH at a 50 mV/s scan rate. Overall, the proposed materials show better electrochemical performance in fuel cell applications.

## Graphical abstract

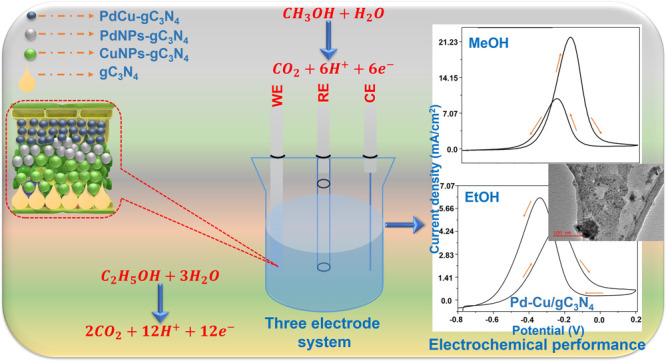

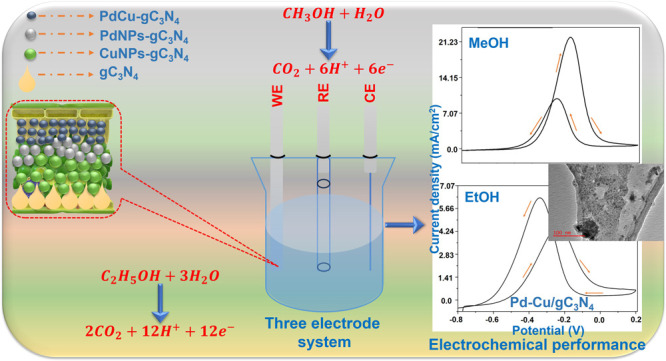

## 1 Introduction

Nowadays, the swift growth of modern computing and wireless digital communication machineries is causing a considerable increase in the demand for power sources for applications demanding less than 250 W. A growing gap between the supply and demand of energy for mobile applications is expected to be filled by fuel cell (FC) devices with increased energy densities.^1^ Due to its two-carbon structure and favorable physical properties, ethanol (EtOH) is the best choice among the alcohols. EtOH is being researched in several fields, including transportation fuels.^2^ Direct ethanol fuel cells (DEFCs) have garnered interest owing to EtOH nontoxic nature in contrast to methanol, coupled with its ability for convenient large-scale production.^3^

Platinum (Pt)-based materials are acknowledged as the premier materials for low-temperature FCs.^4^ Their high cost and susceptibility to catalyst poisoning are evident in these causes, attributed to the development of transitional materials that block the electrocatalytic functionality.^5^ To address these challenges, Pt has been alloyed with various metals to create bimetallic catalysts for the electrooxidation of alcohols, which are utilized as anode materials in energy-generating devices.^6^ However, the limited availability of resources also contributes to the limitations of using Pt-based electrocatalysts. Palladium (Pd) is a more abundant material on Earth than is Pt. In low-temperature FCs, Pd-based materials have also been utilized for formic acid electrooxidation and the oxygen reduction reaction (ORR).^7^

Due to the advantages offered by bimetallic catalysts, different Cu-based bimetallic systems with different components, namely, Cu–Rh,^8^ Cu–Zn,^9^ Cu–Ni,^10^ and Cu–Ag,^11^ have been synthesized for electrocatalytic implementations. Particularly, in various electrochemical reactions, Cu–Pd bimetallic materials have recently outperformed their monometallic counterparts in electrocatalytic activity. The primary source of an improvement in electrocatalytic reaction activity is the geometrical^12^ and electronic^13^ effects, which are produced through the synergistic interface of Cu–Pd bimetals.^14^ The geometric consequence includes changes in the atomic spacing in bimetallic alloys and the three-dimensional distribution of active positions on catalyst surfaces.

Additional noble metal, Pd, is employed as an electrode material with various substrate materials in place of Pt since it is more readily available, less expensive, and has better electrocatalytic activity while being more resistant to carbon monoxide poisoning.^15^ Furthermore, Pd/C exhibits an ORR activity typically five times lower than Pt/C because Pd’s surface forms stronger bonds with oxygenated species than Pt’s. Several techniques were used to increase the ORR catalytic performance of Pd catalysts, including facet-controlled manufacturing of PdNPs, alloying with other metals to create bimetallic composites by using the coreduction method,^16^ and supporting PdNPs (e.g., Pd–Co, Pd–Ni, Pd–Cr) on various support materials like carbon catalysts, which shows better catalytic activity compared to pure Pd because alloying these metal reduce the PdO and overall increases its ORR activity.^17^ A PdNP catalyst supported by carbon is becoming increasingly popular as an anode and cathode electrode for FC applications. Because under FCs’ working conditions, the carbon support is easily electrochemically oxidized into carbon dioxide, which degrades their structure, resulting in the nanoparticles being detached from the surface and showing long-term durability.^18^ gC_3_N_4_ is a 2D material with a structure like graphene. It is a promising support material because it shows good stability, a large surface area, and the ability to interact well with metal NPs.^19^ The high catalytic activity of Pd–CNx is mainly due to a strong interaction between PdNPs and the CNx support due to a downshift in the d-band center of Pd. This electronic shift weakens the adsorption of reaction intermediates (such as CO), improving the overall catalytic efficiency by enhancing reaction kinetics and reducing metal catalyst poisoning.

This work shows how to synthesize gC_3_N_4_-based bimetallic Pd–Cu and use them as catalysts on the glassy carbon electrode (GCE) for effective DEFC and DMFC applications. Several characterization methods demonstrated the successful doping of CuNPs and PdNPs on the gC_3_N_4_ support.

## 2 Experiments

### 2.1 Materials and Methods

In this experiment, analytical-grade chemicals and solvents were utilized in their entirety. All the compounds and substances, for example, potassium tetrachloropalladate­(II) (K_2_PdCl_4_, ≥ 99%), ethanol (EtOH, ≥ 99%), sodium borohydride (NaBH_4_, ≥ 99), urea ((NH_2_)_2_CO, ≥ 98%), ultrapure deionized water (dH_2_O, D.I., 18.25 MΩ), copper sulfate pentahydrate (CuSO_4_·5H_2_O (s), ≥ 98%), were also attained from Merck. These compounds were utilized without further purification.

TEM (JEOL, Tokyo, Japan) tool equipped with a LaB_6_ electron source was used to conduct microscopy studies of the produced material. A small amount of the prepared composite was placed on a 200-mesh Cu grid with a lacy carbon coated to synthesize the material for the TEM study. The XRD patterns were recorded using a Philips PAN analytical X’ pert PRO X-ray diffractometer operating at 40 kV with Cu–Kα radiation (k = 0.1542 nm) over the diffraction angle range 2θ. An fourier transform infrared spectroscopy (FTIR) (IRSpirit QATR-S; A224057 00293-Shimadzu, Kyoto, Japan) analysis was carried out to identify the composite formation. The UV–vis spectrum was recorded using a spectrophotometer (UV 2600; A118757-Shimadzu, Japan) and a quartz cuvette.

The experimentation employed a three-electrode scheme by using a Shanghai Chenhua 760 E potentiostat for electrochemical analysis, with the working electrode (WE), i.e., GCE, where the prepared materials are deposited using the drop and dry method. As a contrast, the Hg/HgO electrode (1 M NaOH) was utilized as the reference electrode (RE), and the Pt electrode was utilized as the counter electrode (CE). The EIS analysis was showed at open-circuit voltage with frequency arrays from 1 MHz to 100 mHz. The prepared catalyst was dried under vacuum at 60 °C and utilized for the X-ray diffraction (XRD) analysis. The obtained catalyst was also confirmed as a suitable anode catalyst for the EtOH and MeOH oxidation reaction.

### 2.2 Electrode Preparation

Three probes were used in the electrochemical design: a counter electrode (CE; Pt wire), an anode electrode, i.e., glassy carbon electrode (diameter of 3 mm), and a RE (Hg/HgO). Using a simple method, the synthesized composites were added one at a time to the WE (GCE), and the electrode was cleansed and cleaned after each cycle. After being washed, the prepared material was employed for the GCE or the probe for the ensuing electrochemical study. Every analysis based on electrochemistry was carried out in a cell that is enclosed with a lid. Electrochemical rinsing with several sizes of alumina solutions and ultrasonication was employed to clean the WE surface. Ultimately, cathodic cleaning and rapid etching with aqua regia were used to verify that the GCE exterior was regenerated. The CE was red-hot from a flame and then cleaned with deionized water. Lastly, while not in use, the RE was stored in base media (1 M NaOH). This phase involved putting the cleaned GCE into an electrochemical apparatus with a PBS (pH 7.5) solution and doing a standard CV investigation at a sweep rate of 50 mV/s in the voltage array of −0.1 V to +0.65 V. The electrochemical scan revealed no redox peaks, indicating that the WE was cleansed entirely and ready to be used for the upcoming electrochemical assessment.^20^ The CV and chronoamperometry (CA) were analyzed utilizing a potentiostat/galvanostat apparatus tailored with a Shanghai Chenhua 760 E potentiostat into a single-cell three-probe arrangement inside an alkaline medium (1 M KOH).

### 2.3 Catalyst Preparation

#### 2.3.1 Preparation of gC_3_N_4_

The method described by Sewnet et al.^21^ was used to manufacture gC_3_N_4_ for this experiment. As a precursor, urea (H_2_N-CO-NH_2_) was used to create gC_3_N_4_. The 15 g of urea is heated for 15 g of urea for 3 h at 80 °C in a covered crucible. This step helps remove any moisture and impurities from the urea. The dried urea is then shifted to a muffle furnace and calcined at 550 °C for 4 h. This high temperature likely induces chemical reactions that convert the urea into gC_3_N_4_. To remove the traces of alkaline species, such as ammonia, the resulting yellow powder is cleaned repeatedly with distilled water. Finally, the washed product is dehydrated at 80 °C for 5 h beneath vacuum to remove any remaining moisture.

#### 2.3.2 Synthesis of CuNPs-gC_3_N_4_

A standard experiment involved dispersing 0.5 g of gC_3_N_4_ in 10 mL of water in 25 mL round-bottom flasks. Each flask received a dropwise addition of a 0.1 M solution of CuSO_4_·5H_2_O (2 wt % of Cu). The Cu salt was then reduced by adding 5 mL of 1 × 10^–3^ M NaBH_4_ solution dropwise to the flask. The material was then filtered, cleaned with water, and dried. The resulting substance, CuNPs-gC_3_N_4_, was used as a catalyst for the electrooxidation of MeOH and EtOH after being characterized by using various methods.

#### 2.3.3 Synthesis Process of PdNPs-gC_3_N_4_

In a typical experiment, each 0.5 g of the synthesized material was mixed in 10 mL of water in a 25 mL round-bottom flask. 0.1 M K_2_PdCl_4_ was added dropwise (2 wt % of Pd). To reduce Pd salt, 5 mL of 1 × 10^–3^ M NaBH_4_ solution was added dropwise to the flask. At the end, the material was filtered, washed with water, and dried. The resultant material, PdNPs-gC_3_N_4_, was analyzed utilizing various methods and was useful as a catalyst for the electrooxidation of MeOH and EtOH.

This formula is used for both Pd and Cu 2% calculation

$$\frac{\mathrm{salt}\;\mathrm{weight}\times \mathrm{weight}\%\times \mathrm{supporting}\;\mathrm{matrial}\;({\mathrm{gC}}_{3}N_{4})}{\mathrm{molecular}\;\mathrm{weight}\;\mathrm{of}\;\mathrm{metal}\times 100}$$

From the above formula, we find *A* gram of salt

$$A=\frac{\mathrm{salt}\;\mathrm{weight}\times \mathrm{molarity}\times x\;\mathrm{volume}}{1000}$$

Using the above formula, we can make any molarity solution.

#### 2.3.4 Synthesis of the Pd-Cu/gC_3_N_4_ Catalyst

A 0.5 g portion of the prepared gC_3_N_4_ was mixed in 10 mL of water. A solution of 0.1 M K_2_PdCl_4_ is added dropwise to the dispersion, resulting in a 2 wt % loading of Pd on the carbon nitride. Next, a solution of 0.250 g of CuSO_4_·5H_2_O in 10 mL of water is slowly added to the mixture while stirring continuously. This introduces Cu ions to the catalyst. A solution of 5 mL of 1 × 10^–3^ M NaBH_4_ is then added slowly to the mixture to reduce the Pd salt to Pd metal NPs. After the mixture was filtered and washed with water to remove unreacted compounds, the resultant material, Pd–Cu/gC_3_N_4_, is dried. Pd (II) was consequently reduced to Pd(0), yielding PdNPs. The synthesized Pd–Cu/gC_3_N_4_ material is characterized using various techniques to analyze its structure and properties. Finally, the catalyst is applied for the electrooxidation of EtOH and MeOH, which will likely test its catalytic activity in this reaction. The graphic illustration of our proposed synthesis process is revealed within Scheme 1.

**Scheme 1:**
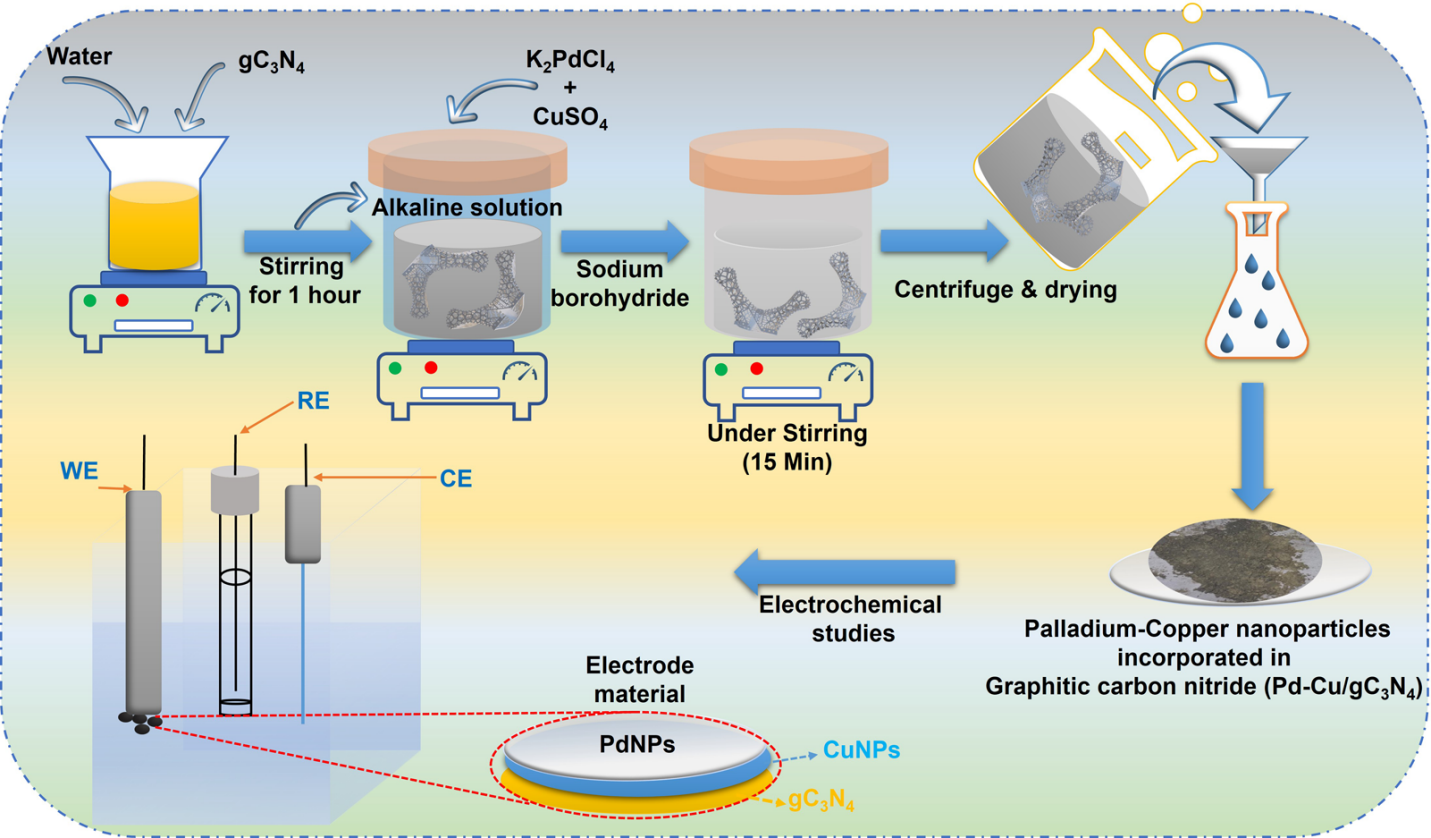
Systematic Diagram of the Proposed Material (Pd-Cu/gC_3_N_4_) Synthesis for FC Application

## 3 Results and Discussion

### 3.1 Microscopic and Optical Characterization

The synthesized materials' phase transparency, i.e., Pd–Cu/gC_3_N_4_, was analyzed using an XRD study, as described within Figure 1(a),(b). XRD detects the phase purity of the prepared nanocomposite. This method indicates whether the prepared material is crystalline or amorphous. The sharp peaks given by the materials are due to their crystalline nature, and the broad peak is due to their amorphous nature. The intense peak at 27.33° showed the formation of gC_3_N_4_,^22^ whereas the strongest peak observed at nearly 40° and the remaining three peaks, 46°, 68°, and 78° confirm the PdNPs formation.^18,23,24^ The peak at 2θ = 36.3° was due to the CuO that was produced in the process of preparation; the other three peaks belong to the planes (111), (200), and (220) of fcc Cu at 2θ = 43.24°, 50.4° and 73.8°, respectively.^25^ The reason was probably that Pd and Cu atoms only partially formed a Cu–Pd alloy structure, and the residual Cu atoms became the Cu NPs.^26^ The gC_3_N_4_ amorphous nature offers superior catalytic activity to improve the catalytic performance of Pd–Cu/gC_3_N_4_. In contrast to pure PdNPs, the samples’ XRD peaks moved to greater 2θ angles, signifying a lattice contraction and alloy production. The integral range approach was used to determine the crystallite size from the XRD data. This method does not consider the dimensions and morphology of crystal domains. This technique gives the Miller indices of the nanocomposite in plane.^27^

**Figure 1:**
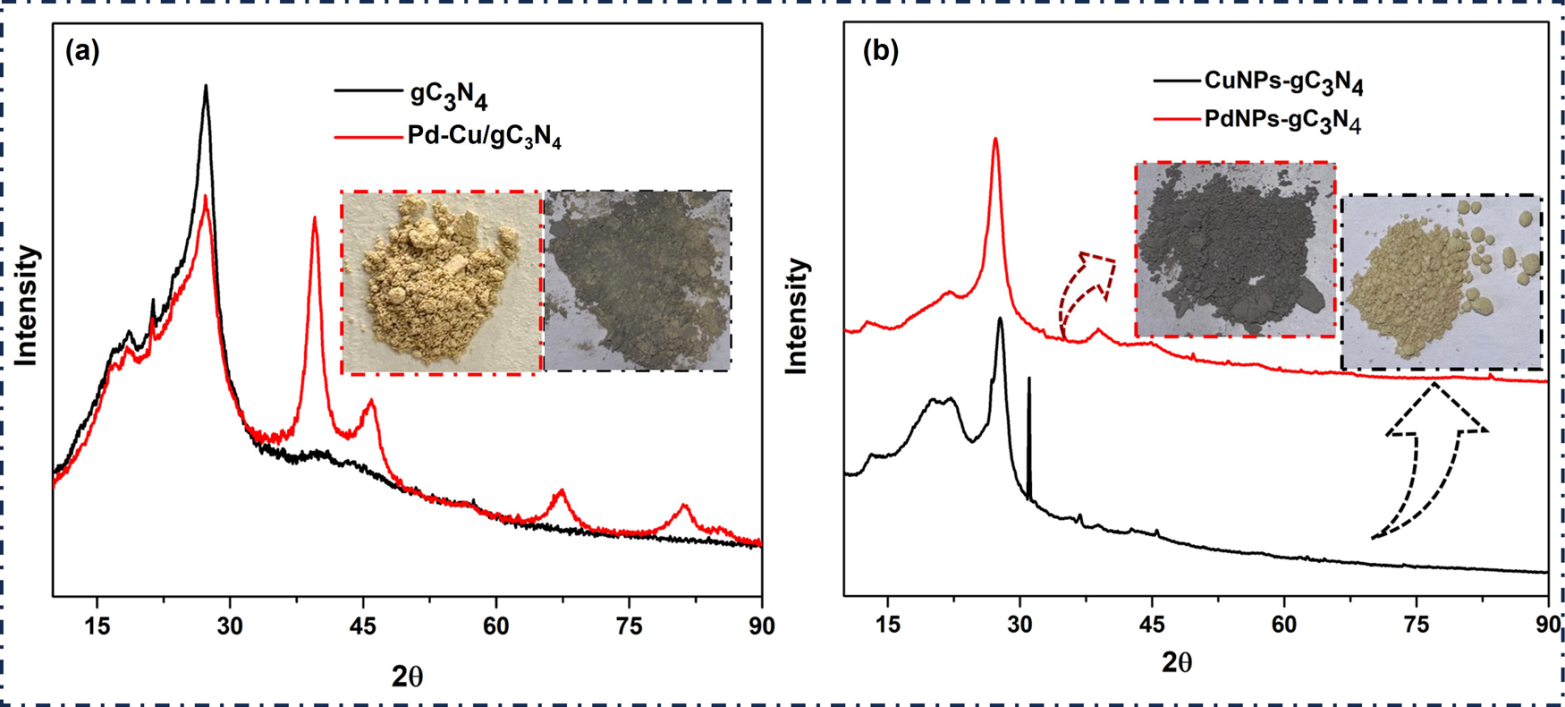
(a) XRD spectra of gC_3_N_4_ (black line) and Pd–Cu/gC_3_N_4_ (red line) and the corresponding optical images. (b) XRD spectra of CuNPs-gC_3_N_4_ (black line) and PdNPs-gC_3_N_4_ (red line) and the corresponding optical images.

The strongest peak at 27.3° paralleled to the (002) planes, usually interplanar stacking assemblies of graphitic substances, as shown in Figure 1(b). The other weak diffraction peak around 13.1° was indexed as (100) planes, the in-plane network filler lattices. It is due to plane-repetitive tri-S-triazine units (interlayer stacking) in gC_3_N_4_ (black line spectra).^28,29^ This is also in line with the XRD result, which shows that when the temperature rises, the 2θ value, which is almost at 27.2°, also increases. This lowers the accompanying *d* value, representing the distance between two subsequent sheets.^30^ The peaks around 22.2°, 38.8°, 46°, and 62.7° are the CuO peaks (black line spectra). The diffraction peaks are strong and fine, demonstrating that the material is highly crystalline and holds a large particle size.^31^ In the PdNPs-gC_3_N_4_ nanocomposite, there are four peaks around 39.92°, 46.43°, 67.76°, and 81.68°, which correspond to the fcc Pd lattices, which match to the (111), (200), (220), and (311) Miller planes, correspondingly.^32^

The morphologies and microstructures of samples were examined by transmission electron microscopy (TEM) and the corresponding particle size distribution histograms of the PdNPs-gC_3_N_4_, CuNPs-gC_3_N_4_, and Pd–Cu/gC_3_N_4_ catalysts are shown as given within Figure 2(a)–(g). TEM provides a flexible substitute for examining fine properties with distinguishing dimensions smaller than 100 nm (or even lower to the atomic range in some cases).^33^ TEM at 200 nm magnification in Figure 2(a) demonstrates the smooth surface and thin film-like structure of gC_3_N_4_. The magnification at 100 nm (Figure 2(b)) shows that the dark spot represents the PdNPs incorporated on the polymeric surface, and the gray shade shows the polymeric gC_3_N_4_. Also, the magnification scale at 200 nm shows that the black dots represent the Cu metal NPs (Figure 2(c)). In Figure 2(d), a magnification scale of 100 nm confirms that our prepared composite (Pd–Cu/gC_3_N_4_) is in the nm range.

**Figure 2:**
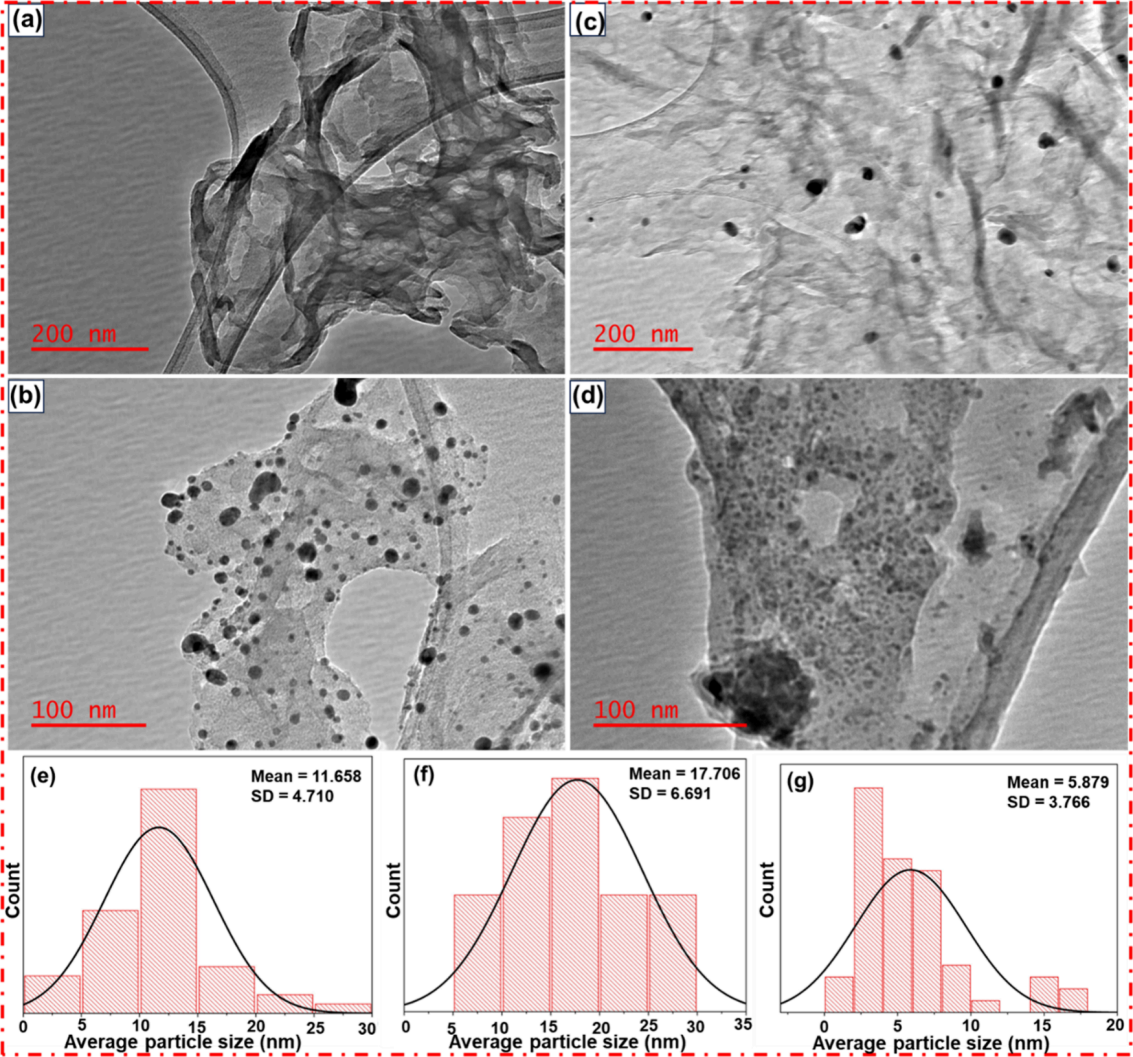
TEM images of (a) gC_3_N_4_, (b) PdNPs-gC_3_N_4_, (c) CuNPs-gC_3_N_4_, and (d) Pd–Cu/gC_3_N_4_. The corresponding particle size distribution histograms of the (e) PdNPs-gC_3_N_4_, (f) CuNPs-gC_3_N_4_, and (g) Pd–Cu/gC_3_N_4_.

As mentioned, evidence of the incorporation of Pd and CuNPs on gC_3_N_4_ was obtained through TEM analysis. Further, we have used energy dispersive X-ray spectroscopy (EDS) for detecting elements present in samples and can additionally quantify the amounts present. Figure 3(a)–(c) shows a typical EDS output. In this case, the spectrum suggests incorporation of Pd and Cu with gC_3_N_4_. The quantification results also confirm the loading of the metals with our proposed material. Two different peaks for Cu and Pd metals are observed in the EDS spectra of the Pd–Cu/gC_3_N_4_ nanocomposite. The distinctive Kα and Kβ emission lines are due to the electronic transitions between different energy levels within the copper and Pd atoms. The nanocomposite structure is indicated by several distinctive peaks, which indicate the integration of Cu and Pd into the gC_3_N_4_.^34^

**Figure 3:**
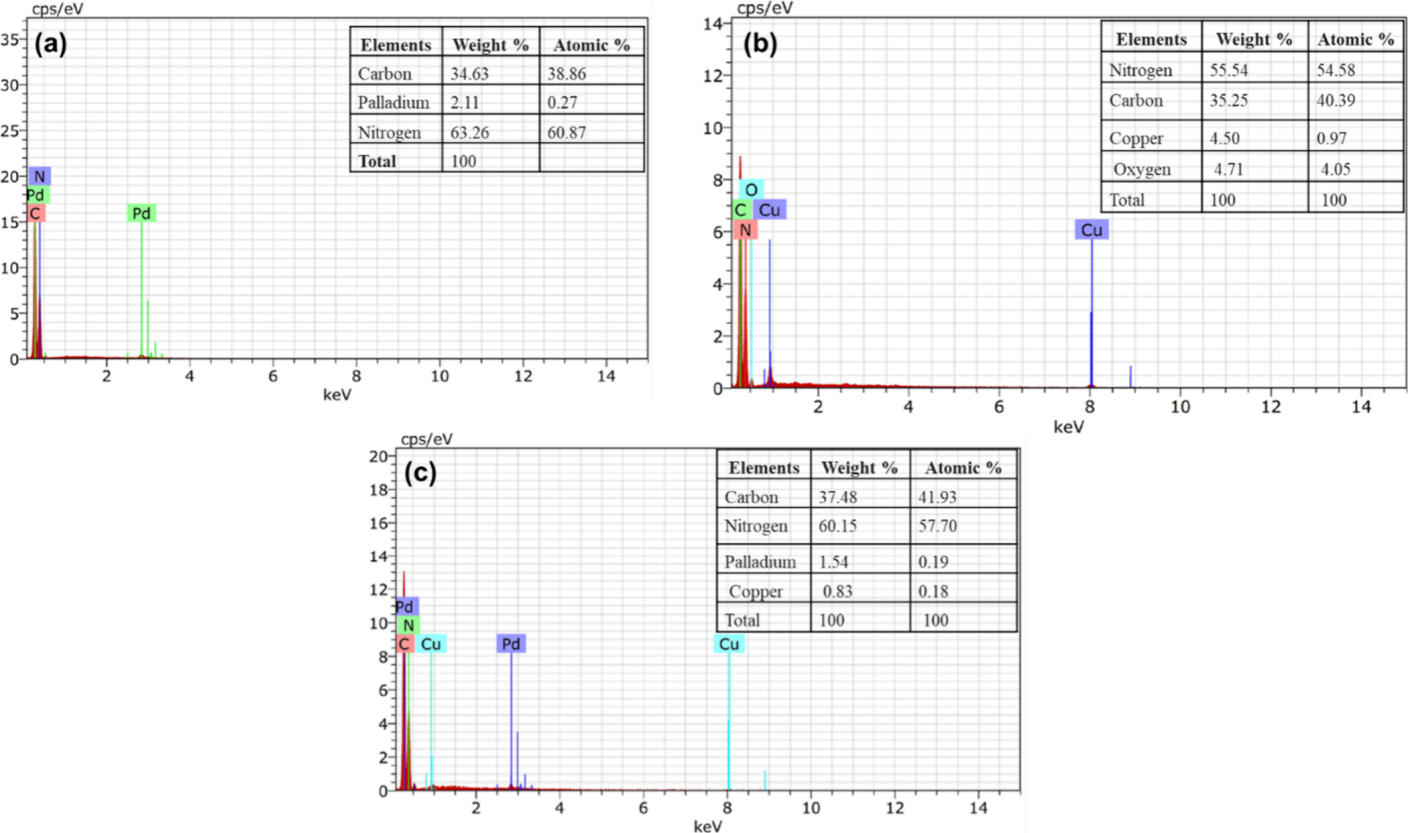
(a–c) EDS elemental spectrum of PdNPs-gC_3_N_4_, CuNPs-gC_3_N_4_, and Pd–Cu/gC_3_N_4_, respectively, and corresponding quantification results.

As shown in Figure 4(a), UV visible spectra of PdNPs-gC_3_N_4_ and CuNPs-gC_3_N_4_ and the corresponding band absorption wavelength further verified the formation of the materials. The UV spectroscopy was performed to identify the result of PdNP doping on gC_3_N_4_ and CuNPs-gC_3_N_4_. The presence of a peak at 380 nm indicates the formation of gC_3_N_4_. As can be seen in Figure 4(b), pure gC_3_N_4_ and Pd–Cu-gC_3_N_4_ displayed an absorption band below 460 nm, which corresponds to its intrinsic band gap transition.^35^ gC_3_N_4_ possesses a bandgap of 2.7 eV, corresponding to an optical wavelength of 460 nm, which depicts it as active under visible light.^36^ When varying concentrations of gC_3_N_4_ were introduced to the CuNPs, it was observed that the absorption peaks were red-shifted from the UV to the visible area.^37^ While PdNPs-gC_3_N_4_ (black curve) exhibits relatively lower absorption intensity across the UV–visible range, the CuNPs-gC_3_N_4_ sample (red curve) shows a higher absorption intensity with distinct shoulders around 310–330 and 420–430 nm. These peaks are observed due to the attributed electronic transition involving the d orbital, as shown in Figure 4(b). These shifts and peak features indicate electronic interactions between Pd and Cu species and the gC_3_N_4_ composite. However, UV–vis spectroscopy only reflects optical absorption and band gap variations; it cannot directly confirm heterojunction formation or photocatalytic efficiency. The nanocomposite exhibits reduced absorbance in the visible spectrum after adding PdNPs.

**Figure 4:**
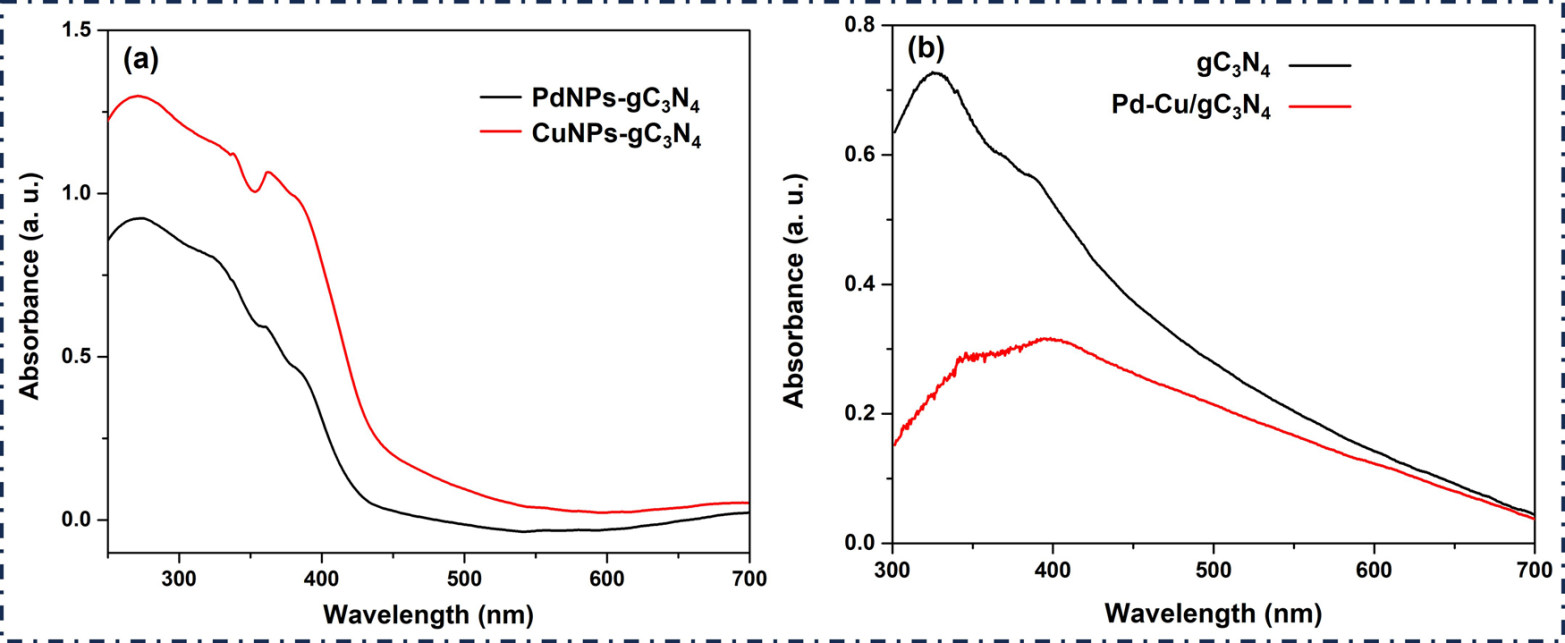
UV visible spectra of (a) PdNPs-gC_3_N_4_ and CuNPs-gC_3_N_4_ where the black curve represents the spectra of PdNPs-gC_3_N_4_ and the red curve indicates CuNPs-gC_3_N_4_. (b) UV–visible spectra of the gC_3_N_4_ (black) and Pd–Cu/gC_3_N_4_ (red) nanocomposite.

A good technique for assessing the lifetime and trapping of electron and hole pairs as well as evaluating the photocatalytic activity is photoluminescence (PL) spectroscopy. After semiconductor irradiation, photon emission from the recombination of e^–^/h^+^ couples causes PL.^38^ The sample exhibits strong PL at room temperature.^39^ The occurrence of PL emissions is largely attributable to band gap transitions and defects generated while preparing the materials. For gC_3_N_4_ and Pd–Cu/gC_3_N_4_, a broad emission peak was noted at approximately 470 nm when excited at 350 nm, that is linked to the band–band PL study, which illustrated in Figure 5.^40^ Both catalysts confirm the PL by a peak value of 470 nm with varying intensities. In comparison to the gC_3_N_4_ and Pd–Cu/gC_3_N_4_ nanocomposite, a relatively lower PL quenching was seen within the Pd–Cu/gC_3_N_4_ material due to partial charge transfer from gC_3_N_4_ to metal NPs.^41^ Incorporating Pd and Cu typically results in PL quenching, indicating an efficient charge separation. The peak is around 470 nm due to the band–band transition, which causes the blue shift observed, which is attributed to band structure modification or defect states introduced by metal incorporation. A band–band PL occurrence of this type is ascribed to excitonic PL, primarily arising from electronic changes concerning the singular pairs of the N atoms in gC_3_N_4_ between the sp^3^ C–N σ–bond and sp^2^ C–N π-bond.^42^ The conjugated network of gC_3_N_4_ has a delocalized electron that causes orbital overlap. Extending the π conjugated system or structural alternation through metal ion doping generates the π* antibonding orbital when more tri-s-triazine rings are joined within gC_3_N_4_. Thus, fast electron transfer can control the gC_3_N_4_ intensity and PL emission peak. With the increase in the doping amount of metal ions, the PL intensity decreases, signifying an enhancement in product crystallinity and a reduction in product defects (Figure 5). This results in fewer exciton recombination centers and leads to a blue shift being observed.^43^

**Figure 5:**
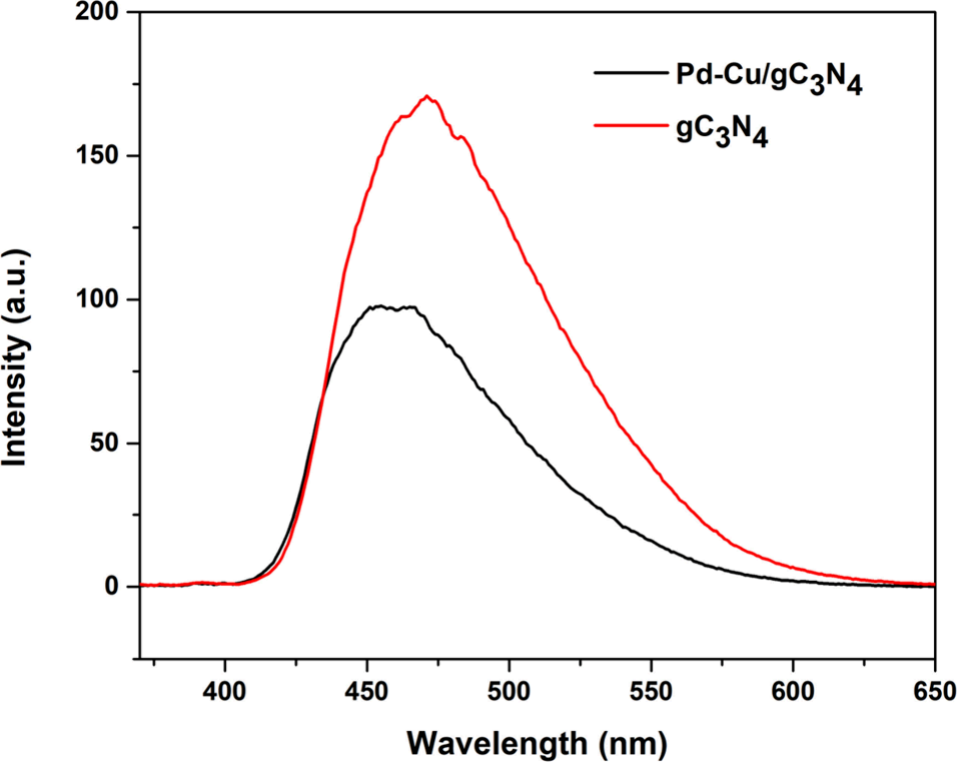
Shows the PL of the prepared gC_3_N_4_ and Pd–Cu/gC_3_N_4_ nanocomposite.

Further, Figure 6(a),(b) shows the FTIR analysis of the prepared materials, which shows the peak around 810–820 cm^–1^, which corresponds to the vibration of the triazine ring and the shifting of this peak owing to the interface with Cu and Pd metal NPs. The peaks around 1200–1650 cm^–1^ are due to the C–N and C=N stretch due to the vibration in the heptazine triazine. This peak occurs at a redshift, while the nitro group is associated with the C=N and C–N stretching vibrations of the heptazine units in gC_3_N_4_. The 450–600 cm^–1^ peaks are due to Pd–N and Cu–N stretching. The peaks at 500–700 cm^–1^ are due to the interaction of metal oxygen with our metals, Pd and Cu. The broad peak at 3000–3500 cm^–1^ is due to N–H stretching due to Cu and Pd metal NP modification, which shows the presence of the amine group. After that, when metal doping occurs on gC_3_N_4_, the shortening of the metal–nitrogen bond takes place, showing the redshift.^24,28,44^ The peak value of 3000–2800 aliphatic group C–H stretching vibrations, the increased intensity of these peaks compared to pure gC_3_N_4_, suggests the successful anchoring of the Pd–Cu NP. Also, this is due to the H content of the complex on the surface of gC_3_N_4_. The Pd–Cu surface may adsorb them more strongly, making the C–H bands more intense.^45^ After modification with Cu and Pd metal ions, all characteristic vibrational peaks related to CuNPs-gC_3_N_4_ (black line spectra) and PdNPs-gC_3_N_4_ (red line spectra) are found in our prepared materials, showing that the structural integrity of our prepared nanocomposite remained intact after doping of the metal ions (Figure 6(b)).

**Figure 6:**
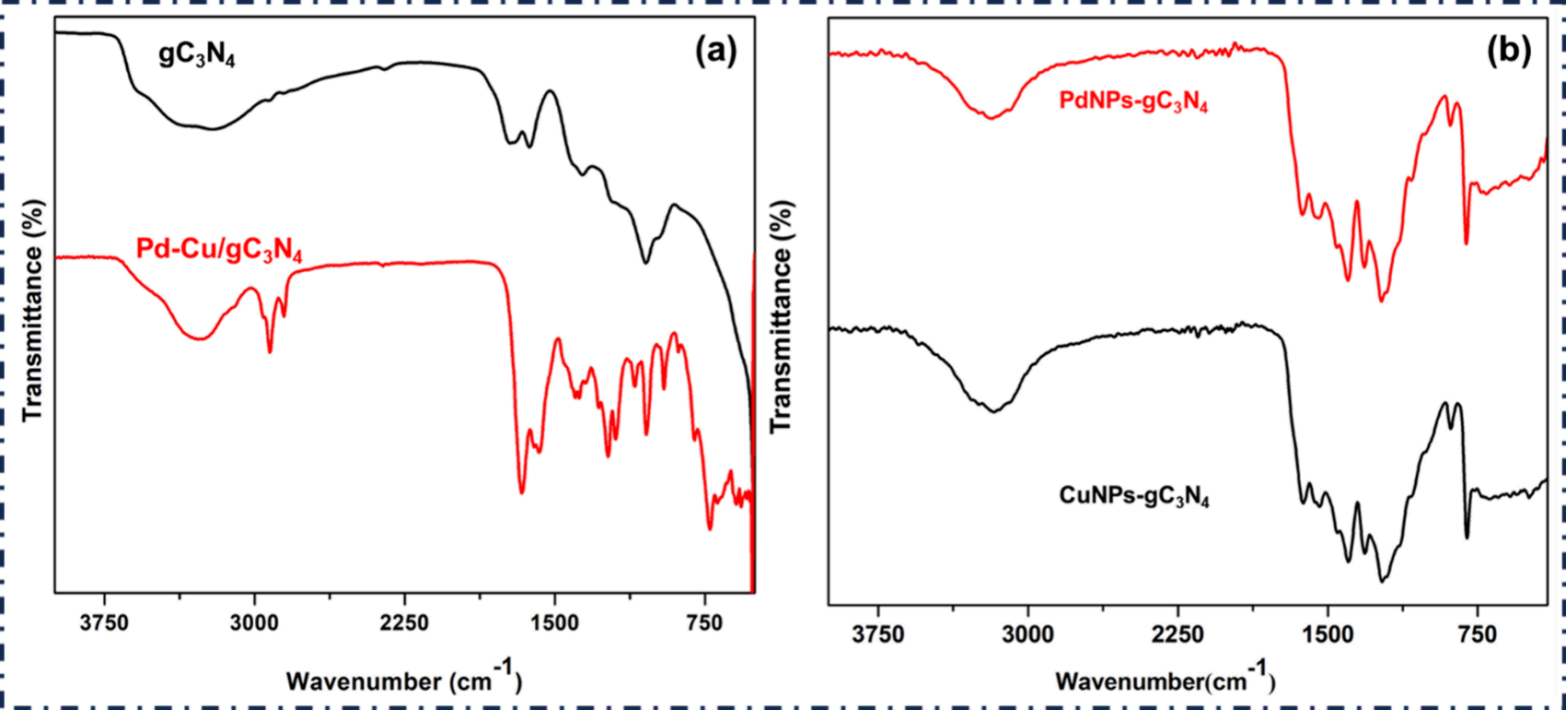
(a) FTIR spectra of gC_3_N_4_ (black line) and Pd–Cu/gC_3_N_4_ (red line). (b) FTIR spectra of CuNPs-gC_3_N_4_ (black line) and PdNPs-gC_3_N_4_ (red line).

### 3.2 Electrochemical Studies

#### 3.2.1 Ethanol

Cyclic voltammetry (CV) is an efficient and broadly utilized electrochemical technique for analyzing molecular species’ reduction and oxidation procedures. CV is a highly helpful technique for studying chemical reactions, such as catalysis reactions, that are started by electron transfer. The speed at which the applied voltage is scanned depends on the experimental scan rate. Faster scan rates result in smaller diffusion layer sizes, which leads to higher current values.^46^ CV measures the current response by oscillating the potential between the defined potential window. The substance’s oxidative and reductive potentials were determined using the reduction and oxidation peaks from the voltammogram. The backward scan has a smaller reduction peak, which is explained by the reduction of intermediate species created during the forward process. However, the forward scan displays a clear oxidation peak corresponding to EtOH electrooxidation. In the CV, an oxidation peak is observed; the forward scan shows a rise in anodic current density (CD).^24^

It is evident from the voltammogram behavior that the addition of alcohol improves the CD for the anode electrode material. Voltammograms for the Pd–Cu/gC_3_N_4_ modified electrodes in KOH (0.5 mol dm^–3^) at a scan rate of 50 mV s^–1^ are shown in the (Figure 7), respectively, with and without EtOH. The maximum CD value for EtOH oxidation obtained for the Pd–Cu/gC_3_N_4_ modified electrode is 4.54 mA cm^–2^ at −0.50 V, indicating the catalytic involvement of Pd and Cu.

**Figure 7:**
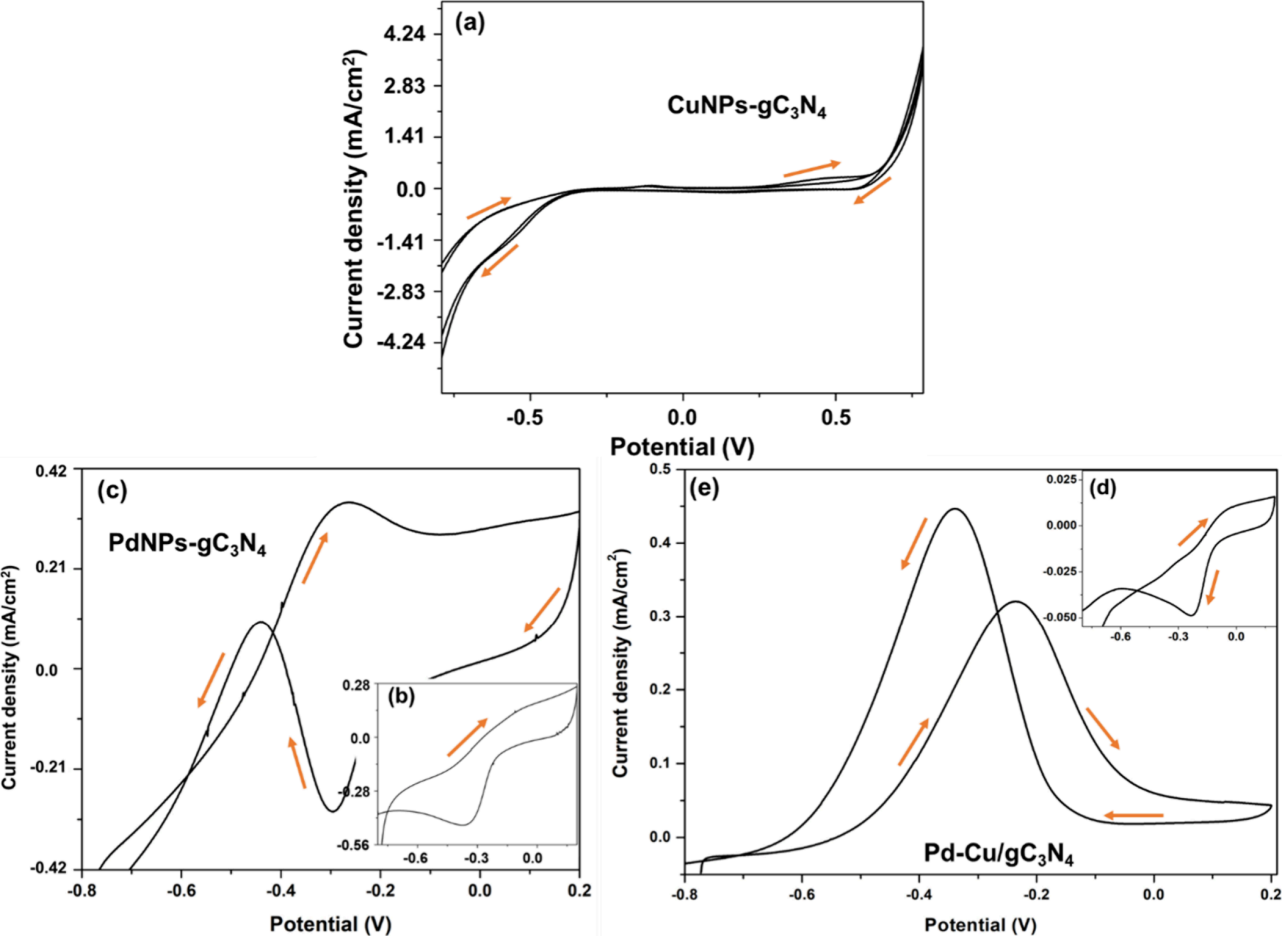
(a) CV of the prepared nanocomposite (CuNPs-gC_3_N_4_) with an analyte (1 M EtOH). (b) CV of the prepared nanocomposite (PdNPs-gC_3_N_4_) without analyte (EtOH). (c) CV of the PdNPs-gC_3_N_4_ nanocomposite with 1 M EtOH, in 0.5 M KOH at the sweep rate 50 mV/s. (d) CV of the prepared nanocomposite (Pd–Cu/gC_3_N_4_) without analyte (EtOH). (e) CV of the Pd–Cu/gC_3_N_4_ nanocomposite with 1 M EtOH, in 0.5 M KOH at the sweep rate 50 mV/s.

Using the CV technique, the material gC_3_N_4_ was examined as a composite with regard to the oxidation of EtOH. Here, Figure 7(a) shows the CV of the prepared nanocomposite (CuNPs-gC_3_N_4_) with an analyte, i.e., EtOH, in a wide potential range. We observed a slight improvement in the CD value with the CuNPs-gC_3_N_4_ nanocomposite. Further, Figure 7(b) shows the CV of the prepared nanocomposite (PdNPs-gC_3_N_4_) without using an analyte, i.e., EtOH. Figure 7(c) displays the CV analysis of the prepared material with EtOH (1M) at a sweep rate of 50 mV/s. The maximum CD is 0.35 mA cm^–2^ at −0.26 V. Figure 7(d) shows the CV of the prepared nanocomposite (Pd–Cu/gC_3_N_4_) without EtOH. Figure 7(e) displays the CV analysis of the prepared material with EtOH (1M) at a sweep rate of 50 mV/s. The highest CD value (4.54 mA cm^–2^) is obtained when we modified the GCE with Pd–Cu/gC_3_N_4_ in the presence of EtOH because complete oxidation of EtOH to CO_2_ provides 12 electrons and 12 protons (complete oxidation pathway) per molecule, which shows EtOH oxidation releases more electrons per molecule (converts EtOH to CO_2_). Especially in the alkaline medium, the CO formation is less, which is the primary motive toward the high catalytic activity of our prepared material and increasing CD.

It is also noteworthy that as peak potential values decrease, a progressive rise in CD values has been noted across the range of scan rates (Figure 8(a)). The EtOH oxidation peaks indicate a slight decrease in potential values and a rise of forward CD values from 2.03 to 6.62 mA cm^–2^. The aforementioned phenomenon may be described in a reaction catalyzed by NPs, in which ionic Pd and Cu transform to a Pd–Cu alloy, producing Pd–Cu/gC_3_N_4_ NPs. The highest CD achieved by using a Pd–Cu/gC_3_N_4_ modified GCE with 1.0 mol dm^–3^ EtOH and 0.5 mol dm^–3^ KOH was 7.79 mA cm^–2^ at −0.221 V (Figure 8(b)). This experiment also examined the Pd–Cu/gC_3_N_4_ modified electrode’s electrocatalytic cycle stability or, to put it another way, the catalyst’s deactivation. The CD value decreased to 6.42 mA cm^–2^ after 100 cycles, indicating partial deactivation as shown in (Figure 8(b)). In short, we conclude that the CV of the catalyst, recorded at different scan rates ranging from 10 to 100 mV/s, is displayed in Figure 8(a) to depict how the scan rate affects the peak CD of ethanol oxidation. On the other side, Figure 8(b) shows the catalyst’s stability test for 100 consecutive cycles, showing strong electrochemical endurance; only a slight decrease in CD indicates good electrochemical durability.

**Figure 8:**
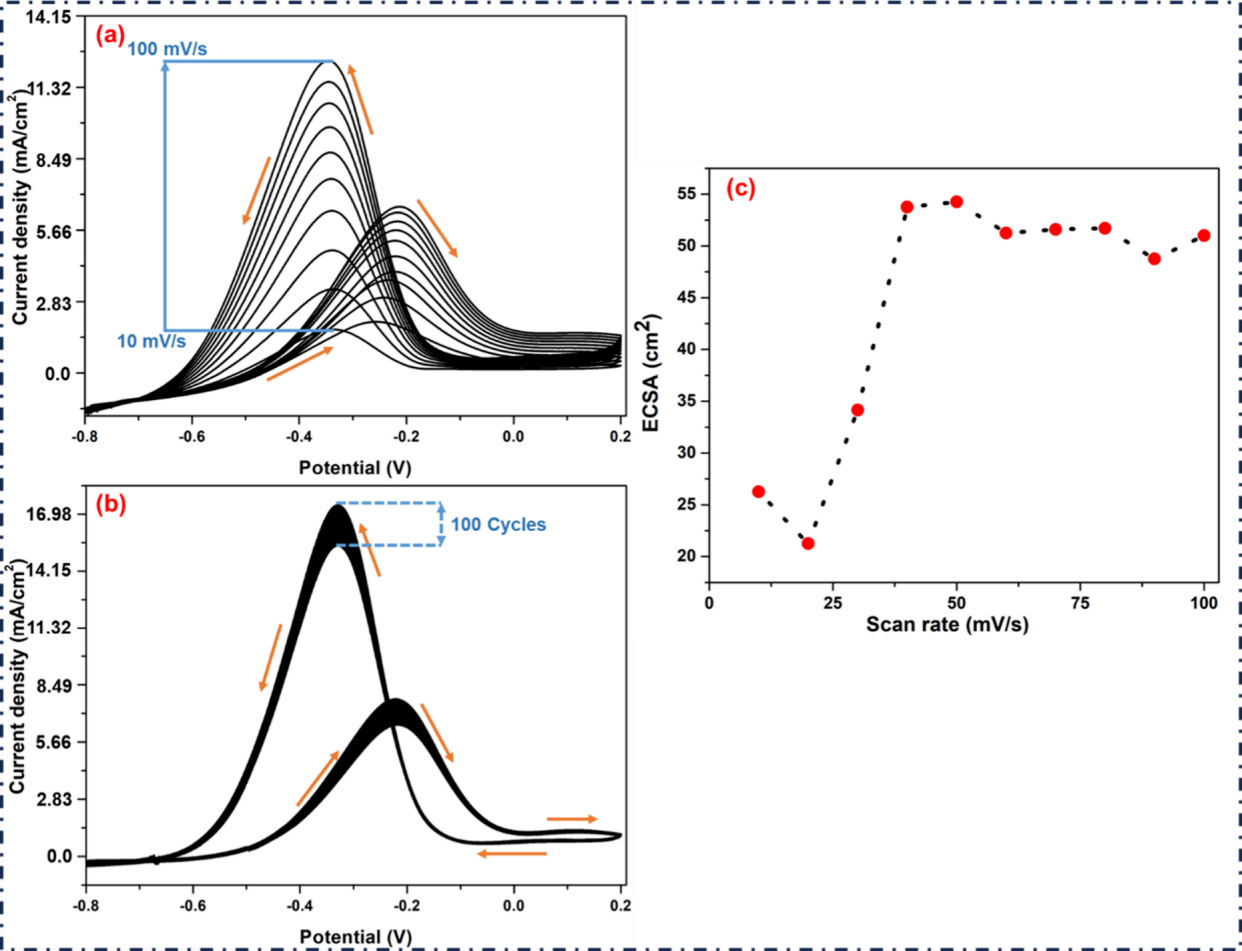
(a) Represents the sweep rate from 10 to 100 mV/s of the CV with 1.0 mol dm^–3^ EtOH and 0.5 mol dm^–3^ KOH. (b) CV of Pd–Cu/gC_3_N_4_ modified GCE shows the stable CD value in the presence of EtOH in 0.5 M KOH at the scan rate of 50 mV/s. (c) Variation of ECSA with scan rate (10–100 mV/s) obtained from CV.

The electrochemically active surface area (ECSA) was determined from CV curves recorded in the region at scan rates ranging from 10 to 100 mV s^–1^. The calculated ECSA values increased from 26.25 cm^2^ at 10 mV s^–1^ to approximately 54 cm^2^ at 50 mV s^–1^ and remained nearly constant up to 100 mV s^–1^. The mean ECSA in this range is 51.4 cm^2^, suggesting a high density of electrochemically accessible active sites that can enhance the catalytic performance in EOR (Figure 8(c)). The equations used to calculate the ECSA are as follows:

$$\left|\delta j=j_{a}-j_{c}\right|$$

$$\frac{\mathrm{scan}\;\mathrm{rate}}{1000}=V/s$$

$$C_{dl}=\frac{\delta j}{2\times \mathrm{scan}\;\mathrm{rate}}\mathrm{mF}/{\mathrm{cm}}^{2}$$

$$\mathrm{ECSA}=\frac{C_{dl}}{C_{s}},\mathrm{where}\;C_{s}=0.04\;\mathrm{mF}/{\mathrm{cm}}^{2}$$

where *C*_*dl*_ is the double layer capacitance, *C_s_* is the specific capacitance, *j_a_* is the anodic CD, and *j_c_* is the cathodic CD.

#### 3.2.2 MeOH

Here, Figure 9 demonstrates the CV of the prepared CuNPs-gC_3_N_4_, PdNPs-gC_3_N_4_, and Pd–Cu/gC_3_N_4_ nanocomposite. Figure 9(a) shows the CV of the prepared nanocomposite (CuNPs-gC_3_N_4_) with the analyte, i.e., MeOH, in a broad potential window. We observed a slight improvement in the CD value for the MeOH electrooxidation reaction with the CuNPs-gC_3_N_4_ nanocomposite in alkaline media. Further, Figure 9(b) displays the CV of the PdNPs-gC_3_N_4_ nanocomposite without analyte methanol (MeOH) and the 0.5 M KOH electrolyte. Figure 9(c) illustrates the CV of the PdNPs-gC_3_N_4_ with a modified GCE with 1 M MeOH and 0.5 M KOH at a sweep rate of 50 mV/s. The maximum CD is 12.05 mA cm^–2^. Figure 9(d) displays the CV of the Pd–Cu/gC_3_N_4_ nanocomposite without analyte (MeOH) and the 0.5 M KOH electrolyte. Figure 9(e) illustrates the CV of the prepared material with a modified GCE with 1 M MeOH and 0.5 M KOH at a sweep rate of 50 mV/s. The maximum CD is 22.16 mA cm^–2^.

**Figure 9:**
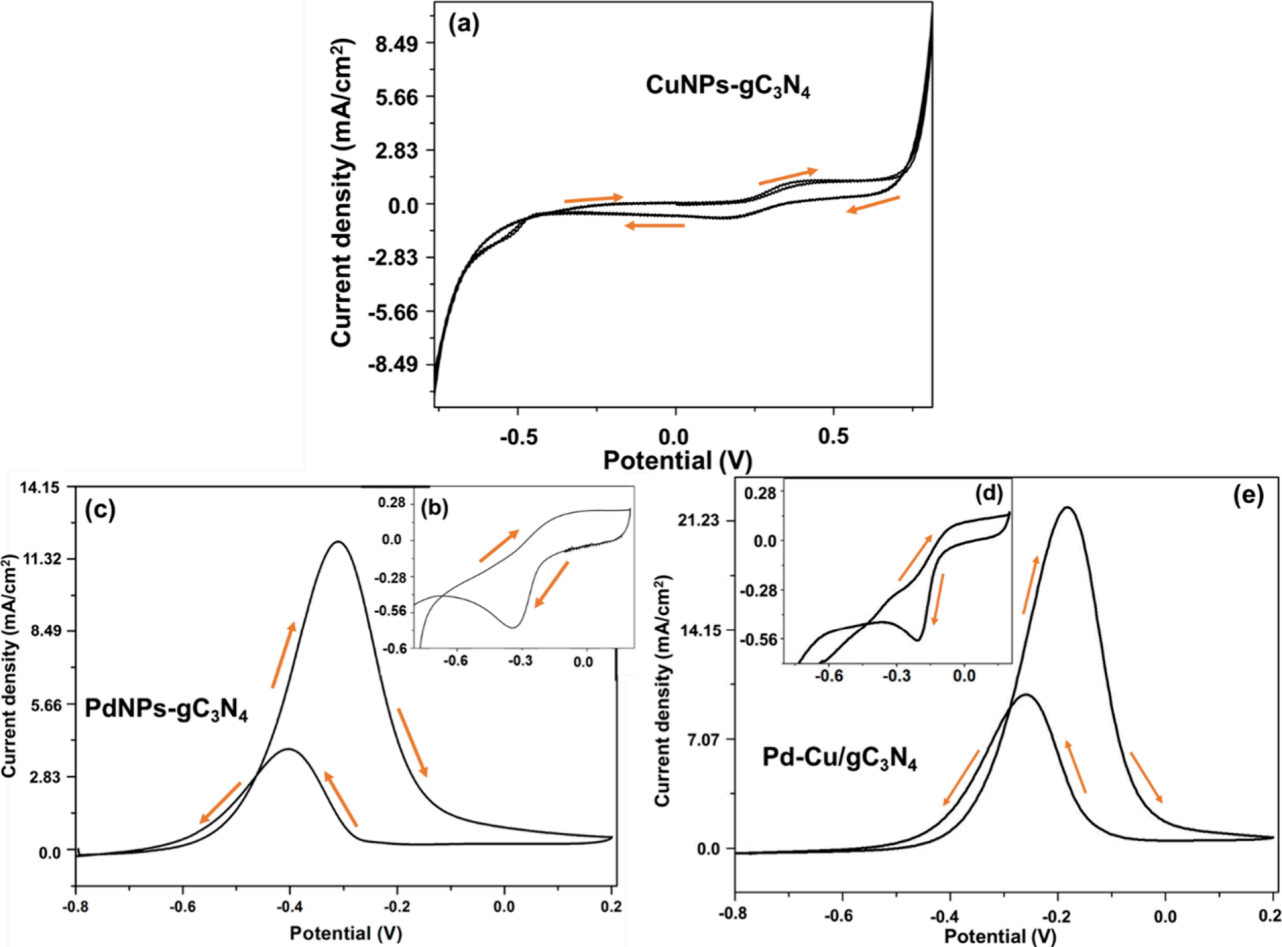
(a) CV of CuNPs-gC_3_N_4_ in the presence of 1 M MeOH. (b) CV of the PdNPs-gC_3_N_4_ in the absence of MeOH. (c) CV of the prepared PdNPs-gC_3_N_4_ with 1 M MeOH with a 0.5 M KOH electrolyte. (d) The prepared material’s CV in the absence of MeOH. (e) CV of the prepared Pd–Cu/gC_3_N_4_ with 1 M MeOH with a 0.5 M KOH electrolyte.

It is also crucial to note that when the peak potential value decreased, a steady rise in CD values was seen across the range of scan rates (Figure 10(a)). The MeOH oxidation peaks indicate a slight decrease in potential values and an increase in forward CD values from 3.84 to 30.92 mA cm^–2^. The aforementioned possibility would be clarified in a catalyzed reaction in which Pd–Cu/gC_3_N_4_ NPs are formed due to the conversion of ionic Pd and Cu to a Pd–Cu alloy. The highest CD achieved by using Pd–Cu/gC_3_N_4_ modified GCE with 1.0 mol dm^–3^ MeOH and 0.5 mol dm^–3^ KOH was 44.44 mA cm^–2^ at −0.172 V (Figure 10(b)). This experiment also examined the electrocatalytic cycling stability of the Pd–Cu/gC_3_N_4_ modified electrode or the deactivation of the catalyst. After 100 scans, we realized that the net decrease in forward CD was 40.62 mA cm^–2^, demonstrating a durable material for the MeOH oxidation reaction (Figure 10(b)). The observed decrease of ECSA with increasing scan rate in MeOH does not mean the real surface area is changing. It indicates that charge transfer and adsorption kinetics are scan-rate dependent (Figure 10(c)).

**Figure 10:**
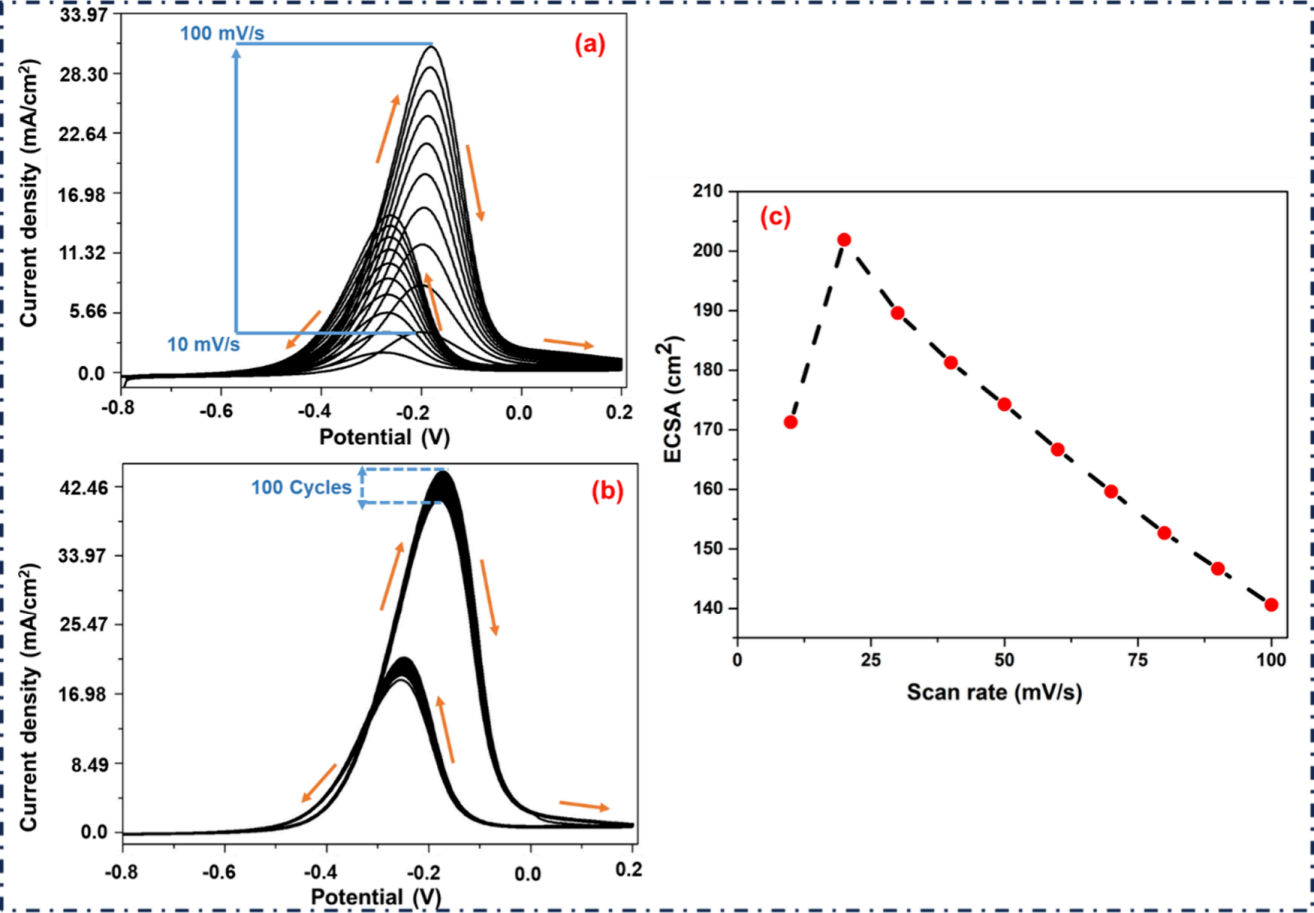
(a) Represents the sweep rate 10 to 100 mV/s of the CV with 1.0 mol dm^–3^ MeOH and 0.5 mol dm^–3^ KOH. (b) CV of the Pd–Cu/gC_3_N_4_-doped GCE shows the CD value at each scan with MeOH in 0.5 M KOH at the sweep rate of 50 mV/s. (c) The variation of ECSA with scan rate (10–100 mV s^–1^) obtained from CV.

Chronoamperometry (CA) is an electrochemical method that measures the current response over time in a potential step in electroanalysis. This process entails applying a potential to an electrode and monitoring the ensuing current over time. The current recorded during CA comprises two distinct components: faradaic and nonfaradaic.^47^ The resulting current arising from applying the step from the reversible potential to a specified overpotential value is recorded within a short period (*t* < 0.01 s) and then plotted against the time. The initial CD at such overpotential is not determined by simply extrapolating the resulting straight line at *t* → 0. It can be determined by extrapolating at *t* → 0, the Cottrell equation, which characterizes the diffusion-controlled decrease in the current as *i*(*t*) ∝ *t* ^–1/2^. This equation was used to study the transient current response. This method provides that the examined parameters accurately represent the electrochemical kinetics of the catalyst system and offers a more accurate interpretation of the charge transport and diffusion behavior during ethanol oxidation.^48^ The curves shown in Figure 11 present chronoamperometric curves showing CD vs time. The chronoamperometric responses for the Pd–Cu/gC_3_N_4_, PdNPs-gC_3_N_4_, CuNPs-gC_3_N_4_, and gC_3_N_4_ catalysts with 1.0 M EtOH and 0.5 M KOH at 30 °C at the static voltage of −0.6 V for 3000 s are shown in Figure 11(a),(b). The highest CD of the final prepared nanocomposite is nearly 21.23 mA/cm^2^, and Pd–Cu/gC_3_N_4_ (black line) shows the best catalytic activity. The performance of PdNPs-*g*-C_3_N_4_ (blue line) is nearly 14.15 mA/cm^2^. The lowest CD of 7.07 mA/cm^2^ is obtained by CuNPs-*g*-C_3_N_4_ (red line), indicating lower catalytic efficiency. The final prepared nanocomposite shows good stability over 3000 s.

**Figure 11:**
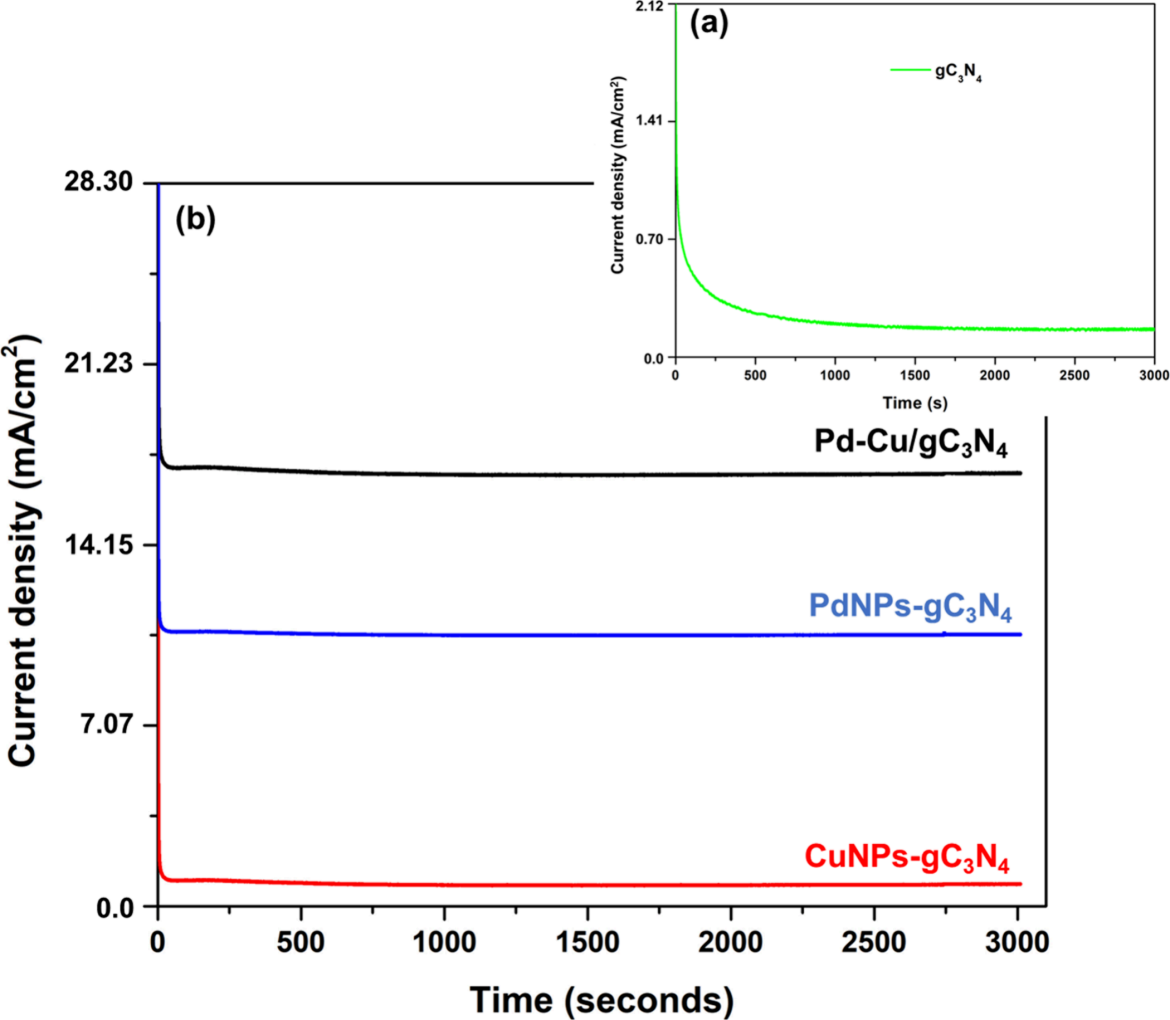
CA of the prepared (a) gC_3_N_4_ and (b) the Pd–Cu/gC_3_N_4_, PdNPs-gC_3_N_4_, and CuNPs-gC_3_N_4_ nanocomposite for 3000 s.

An efficient technique for examining the characteristics of a surface-modified WE is electrochemical impedance spectroscopy (EIS). This technique shows *Z* = *Z*′ + *jZ*″, where *j* is the imaginary unit and the real part and *Z*′ shows real and imaginary (*Z*″) components, which shows that capacitors contribute only to the *Z*″ impedance (*Z*″) and not to component Z′. The resistance arises from factors, such as charge transfer resistance. In the frequency range of 1 MHz to 100 mHz, the Nyquist plot was used to examine the relationship between *Z*′ and *Z*″ part of the impedance for the samples gC_3_N_4_ and CuNPs-gC_3_N_4_ under the alkaline condition (KOH, 0.5 mol dm^–3^), as shown in Figure 12(a). The Nyquist plot in the main panel shows a straight line after a semicircle area on the *Z*′-axis. The electron-transfer-restricted process is represented by the semicircle section, which is seen in a higher frequency region. On the other hand, the diffusion-limited electron-transfer mechanism is represented by the linear portion, which is typical of the lower frequency range. Nearly comparable slope values were obtained for gC_3_N_4_ and CuNPs-gC_3_N_4_, as can be seen from Figure 12(a),(b). This implies that both samples have the same ion diffusion rate. The semicircle diameter in the Nyquist plot indicates the electrode surface electron transfer resistance values, which are 84.26 and 63.47 Ω for gC_3_N_4_ and CuNPs-gC_3_N_4_ correspondingly.

**Figure 12:**
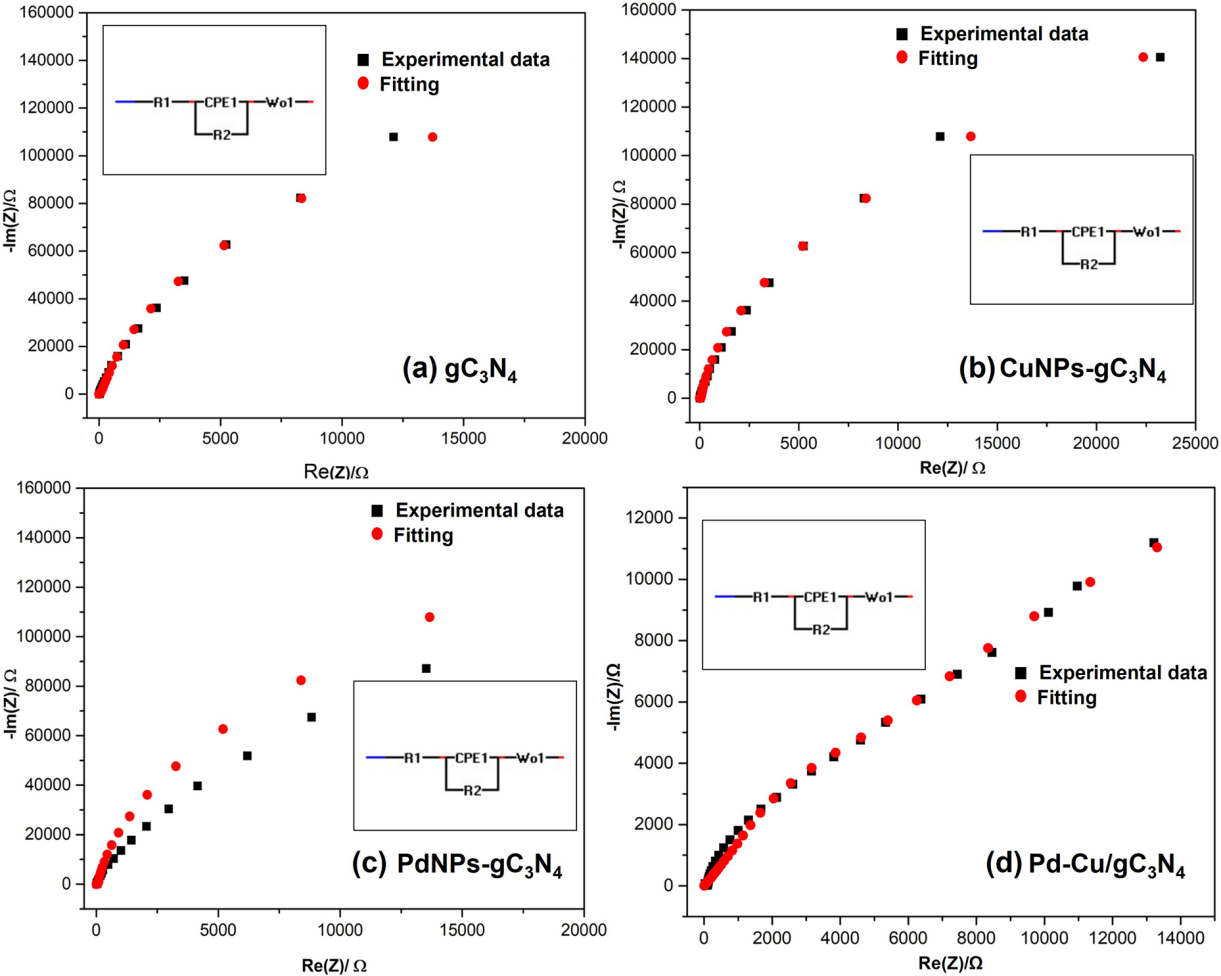
EIS for gC_3_N_4_ (a) and CuNPs-gC_3_N_4_ (b) with the *R*_ct_ value of 84.26 and 63.47 Ω, respectively. PdNPs-gC_3_N_4_ (c) and Pd–Cu/gC_3_N_4_ (d) with the *R*_ct_ value of 17.91 and 14.67 Ω, respectively. All of the measurements are in the frequency range from 1 MHz to 100 mHz.

The high electron transfer resistance value (84.26 Ω) for the gC_3_N_4_ sample suggests that the polymer acts as a kinetic barrier during the electron transfer process. Due to the presence of CuNPs, which improved the charge transfer mechanism, the electron transfer resistance value for the CuNPs-gC_3_N_4_ sample dropped to 63.47 Ω. The Nyquist plots of the PdNPs-gC_3_N_4_ and Pd–Cu/gC_3_N_4_-doped electrodes are shown in Figure 12(c),(d). The measurements were made in KOH (0.5 mol dm^–3^) between 1 MHz and 100 mHz. The enlarged impedance spectra suggest a reduced ohmic loss in the presence of KOH electrolyte at higher frequencies because of its high ionic conductivity and show the efficient transport of OH^–^ ions, which lowers internal resistance within the electrochemical cell. The high electron transfer resistance measurement (17.91 Ω) for the PdNPs-gC_3_N_4_ sample suggests that the supporting material acts as a kinetic barrier to the electron transfer process. Adding CuNPs to the sample Pd–Cu/gC_3_N_4_ resulted in a lower electron transfer resistance value (14.67 Ω), enhancing the charge transfer mechanism.

Table 1shows the electrochemical performance of different materials for EtOH and MeOH oxidation reactions.

**Table 1:** Electrochemical Performance of Different Materials for EtOH and MeOH Oxidation Reactions

electrochemical performance for EtOH oxidation				
catalyst	electrolyte	analyte	CD (mA/cm^2^)	references
PdNi/C	1 M NaOH	1 M EtOH	0.96	^49^
PdNiSn/C	1 M NaOH	1 M EtOH	3.14	^49^
Pd–Ni/Carbon nanofiber	0.5 M NaOH	1 M EtOH	1.18	^50^
Pt/C	0.5 M NaOH	1 M EtOH	0.0003	^50^
Pd-Cu/gC_3_N_4_	0.5 M KOH	1 M EtOH	4.54	present work
**electrochemical performance for MeOH oxidation**				
Co/Cu-decorated Carbon NFs	1 M KOH	2 M MeOH	17.0	^51^
NiCo_2_O_4_-RGO	0.1M	0.5 M MeOH	0.05	^52^
PdNPs-ZnO-gCN	0.5 M KOH	0.5 M MeOH	0.96	^53^
Pd-Cu/gC_3_N_4_	0.5 M KOH	1 M MeOH	22.16	present work

## 4 Conclusions

In this research, the prepared electrode catalysts demonstrate good performance for EtOH and MeOH FC applications. The materials based on gC_3_N_4_ and Cu-modified Pd–Cu/gC_3_N_4_ were prepared with approximately 2% CuSO_4_ by weight and 2% K_2_PdCl_4_. All of the prepared catalysts were utilized as anode materials for modifying GCE electrodes to facilitate EtOH and MeOH oxidation within an alkaline environment. Experimental results indicate that incorporating CuNPs and PdNPs into gC_3_N_4_ enhances the electrooxidation of EtOH and MeOH by augmenting the superior catalytic activity of the supporting materials, namely gC_3_N_4_. The catalyst encouraging electrochemical performance increases the possibility of future beneficial uses, but more research into scalability and long-term durability is required to determine its commercial feasibility. The presence of the crystalline metallic PdNPs in the gC_3_N_4_ was confirmed by the microscopic and surface characterization techniques. In alkaline media, the Pd–Cu/gC_3_N_4_ nanocomposite’s integrated architecture exhibits good durability against electrocatalytic EtOH and MeOH oxidation.

## Abbreviation

FC: Fuel cell
EtOH: Ethanol
MeOH: Methanol
DEFCs: Direct ethanol fuel cells
Pt: Platinum
Pd: Palladium
ORR: Oxygen reduction reaction
gC_3_N_4_: Graphitic carbon nitride
GCE: Glassy carbon electrode
CE: Counter electrode
WE: Working electrode
RE: Reference electrodes
CV: Cyclic voltammetry
CA: Chronoamperometry
XRD: X-ray diffraction
CD: Current density
SA: Surface area
nm: Nanometer
TEM: Transmission electron microscopy
PL: Photoluminescence
TMs: Transition metals
NPs: Nanoparticles
Cu: Copper
EDS: Energy dispersive X-ray spectroscopy
ECSA: Electrochemical active surface area
